# Side-Chain Modified [^99m^Tc]Tc-DT1 Mimics: A Comparative Study in NTS_1_R-Positive Models

**DOI:** 10.3390/ijms242115541

**Published:** 2023-10-24

**Authors:** Panagiotis Kanellopoulos, Berthold A. Nock, Maritina Rouchota, George Loudos, Eric P. Krenning, Theodosia Maina

**Affiliations:** 1 Molecular Radiopharmacy, INRaSTES, NCSR “Demokritos”, 15341 Athens, Greece; kanelospan@gmail.com (P.K.); nock_berthold.a@hotmail.com (B.A.N.); 2 BIOEMTECH, Lefkippos Attica Technology Park NCSR “Demokritos”, 15310 Athens, Greece; mrouchota@bioemtech.com (M.R.); george@bioemtech.com (G.L.); 3 Cyclotron Rotterdam BV, Erasmus MC, 3015 CE Rotterdam, The Netherlands; erickrenning@gmail.com

**Keywords:** neurotensin subtype 1 receptor, radiolabeled neurotensin, lateral chain modification, diagnostic imaging, Tc-99m-radioligand, metabolic stability, neprilysin, angiotensin-converting enzyme, peptidase inhibition

## Abstract

Radiolabeled neurotensin analogs have been developed as candidates for theranostic use against neurotensin subtype 1 receptor (NTS_1_R)-expressing cancer. However, their fast degradation by two major peptidases, neprilysin (NEP) and angiotensin-converting enzyme (ACE), has hitherto limited clinical success. We have recently shown that palmitoylation at the *ε*-amine of Lys^7^ in [^99m^Tc]Tc-[Lys^7^]DT1 (DT1, N_4_-Gly-Arg-Arg-Pro-Tyr-Ile-Leu-OH, N_4_ = 6-(carboxy)-1,4,8,11-tetraazaundecane) led to the fully stabilized [^99m^Tc]Tc-DT9 analog, displaying high uptake in human pancreatic cancer AsPC-1 xenografts but unfavorable pharmacokinetics in mice. Aiming to improve the in vivo stability of [^99m^Tc]Tc-DT1 without compromising pharmacokinetics, we now introduce three new [^99m^Tc]Tc-DT1 mimics, carrying different pendant groups at the *ε*-amine of Lys^7^: MPBA (4-(4-methylphenyl)butyric acid)—[^99m^Tc]Tc-DT10; MPBA via a PEG4-linker—[^99m^Tc]Tc-DT11; or a hydrophilic PEG6 chain—[^99m^Tc]Tc-DT12. The impact of these modifications on receptor affinity and internalization was studied in NTS_1_R-positive cells. The effects on stability and AsPC-1 tumor uptake were assessed in mice without or during NEP/ACE inhibition. Unlike [^99m^Tc]Tc-DT10, the longer-chain modified [^99m^Tc]Tc-DT11 and [^99m^Tc]Tc-DT12 were significantly stabilized in vivo, resulting in markedly improved tumor uptake compared to [^99m^Tc]Tc-DT1. [^99m^Tc]Tc-DT11 was found to achieve the highest AsPC-1 tumor values and good pharmacokinetics, either without or during NEP inhibition, qualifying for further validation in patients with NTS_1_R-positive tumors using SPECT/CT.

## 1. Introduction

The neurotensin subtype 1 receptor (NTS_1_R) has attracted considerable attention in oncology [1,2] by virtue of its high-density expression in a variety of human tumors, including pancreatic [3,4,5] and colorectal cancer [6], Ewing’s sarcoma [7], prostate [8] and breast carcinoma [9]. Following a “radiotheranostic” concept, NTS_1_R may serve as a valid biomolecular target for directing neurotensin (NT, pyroGlu-Leu-Tyr-Glu-Asn-Lys-Pro-Arg-Arg-Pro-Tyr-Ile-Leu-OH)-based radioligands to tumor lesions for either diagnostic imaging or the delivery of radiotoxic payloads, depending upon the radionuclide employed [10,11,12,13]. A good number of radioligands based on NT and its terminal NT (8–13) fragment have been developed for theranostic use against NTS_1_R-positive cancers [10,11,14,15,16,17,18], but hitherto clinical progress has been restricted by their fast degradation in the biological milieu [19,20,21]. Amongst the peptidases breaking down NT and its analogs in the body, neprilysin (NEP) and angiotensin-converting enzyme (ACE) have been shown to play a leading role [22,23,24,25]. Recently, we were able to confirm this hypothesis in the case of [^99m^Tc]Tc-DT1 (DT1, N_4_-Gly-Arg-Arg-Pro-Tyr-Ile-Leu-OH, N_4_= 6-(carboxy)-1,4,8,11-tetraazaundecane) and its analogs. Following the administration of suitable NEP/ACE inhibitors, we induced a significant enhancement of radioligand stability in mice circulation, which advantageously translated into marked improvements in uptake in NTS_1_R-expressing tumors in mice [26,27].

The in situ inhibition of ACE, otherwise known as peptidyl dipeptidase A, cutting-off C-terminal dipeptides from NT and other substrates [24,25,28], could be achieved using lisinopril (Lis). The latter is a potent ACE inhibitor that is used as an antihypertensive drug [29]. Likewise, in situ NEP inhibition could be achieved by potent and selective inhibitors released in vivo following the administration of registered formulations containing a pro-drug, such as the anti-diarrhea Hidrasec^®^ capsules or granules (releasing thiorphan from the precursor racecadotril) [30,31,32] or the antihypertensive drug Entresto^®^ (releasing sacubitrilat from the precursor sacubitril) [33,34,35]. In view of the regulatory and biosafety concerns linked to the co-administration of NEP and ACE inhibitors in patients, we have recently attempted a series of C-terminal structural interventions on [^99m^Tc]Tc-DT1 to improve its metabolic stability. The resulting [^99m^Tc]Tc-DT1 mimics, despite their better resistance to ACE, turned out to be less effective with regards to cell internalization or tumor targeting in mice, even during ACE/NEP inhibition regimens [26,27,36]. Unlike the C-terminal modified radioligands, [^99m^Tc]Tc-DT9 carrying a palmitoyl chain attached to Lys^7^ (replacing Gly^7^ in the original DT1 structure) acquired full resistance to ACE and NEP in peripheral mice blood and a high uptake in human pancreatic cancer AsPC-1 xenografts in mice [36]. These positive qualities, however, were compromised by the high background radioactivity levels and the overall unfavorable pharmacokinetic profile of [^99m^Tc]Tc-DT9. Palmitoylation has been applied to increase the in vivo stability and bioavailability of several peptide analogs [37,38,39,40]. Interestingly, it has led to improved analogs of contulakin-G, a 16-mer peptide isolated from the venom of the sea snail *Conus geographus* (pGlu-Ser-Glu-Glu-Gly-Gly-Ser-Asn-Ala-Thr(R)-Lys-Lys-Pro-Tyr-Ile-Leu-OH), with a disaccharide covalently attached to Thr^10^ [41]. Previous studies have revealed that this residue corresponds to Pro^7^ in NT and that substitutions/the attachment of side chains in either peptide has yielded analogs with a higher stability and affinity for the human NTS_1_R [42,43], thus providing the rationale for the design of [^99m^Tc]Tc-DT9 [36].

**Figure 1:**
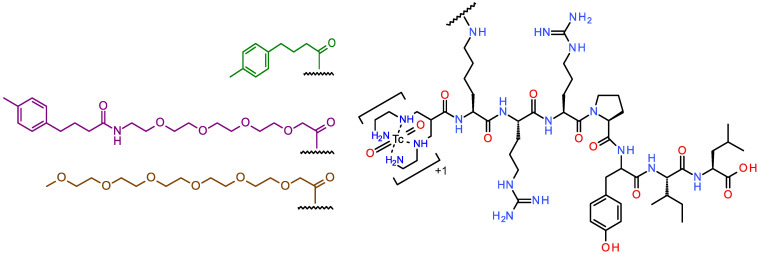
Chemical structures of [^99m^Tc]Tc-DT1 (DT1, N_4_-Gly^7^-Arg-Arg-Pro-Tyr-Ile-Leu-OH; N_4_, 6-(carboxy)-1,4,8,11-tetraazaundecane) mimics, carrying different pendant groups attached to the *ε*-amine of Lys^7^: [^99m^Tc]Tc-DT10 (green, MPBA = 4-(4-methylphenyl)butyric acid); [^99m^Tc]Tc-DT11 (violet, MPBA-PEG4, PEG4: 14-amino-3,6,9,12-tetraoxatetradecan-1-oic acid); and [^99m^Tc]Tc-DT12 (brown, mPEG6-CH_2_-COOH: 2,5,8,11,14,17-hexaoxanonadecan-19-oic acid).

Aiming for improvements in the in vivo stability of [^99m^Tc]Tc-DT1 without compromising its pharmacokinetics, we herein introduce three new [^99m^Tc]Tc-DT1 mimics, carrying different pendant groups at the *ε*-amine of Lys^7^: (i) [^99m^Tc]Tc-DT10 directly coupled to the albumin-binding domain (ABD) MPBA (4-(4-methylphenyl)butyric acid) [44,45], (ii) [^99m^Tc]Tc-DT11 carrying the MPBA group attached via a PEG4-linker (14-amino-3,6,9,12-tetraoxatetradecan-1-oic acid), and (iii) [^99m^Tc]Tc-DT12 with a non-ABD hydrophilic PEG6 chain (mPEG6-CH_2_-COOH = 2,5,8,11,14,17-hexaoxanonadecan-19-oic acid) [46], as presented in Figure 1. The impact of these modifications on receptor affinity and internalization was studied in NTS_1_R-positive cells, whereas the effects on metabolic stability and AsPC-1 tumor uptake were assessed in mice without or during NEP/ACE inhibition in comparison with the unmodified [^99m^Tc]Tc-DT1 reference. We were especially interested in investigating the contribution of albumin binding (ABD-containing [^99m^Tc]Tc-DT10 and [^99m^Tc]Tc-DT11) vs. steric factors ([^99m^Tc]Tc-DT11 and [^99m^Tc]Tc-DT12 carrying lateral chains of comparable length to palmitoyl).

## 2. Results

### 2.1. Ligands and Radioligands

The analytical data for the DT1 mimics DT10, DT11 and DT12, carrying diverse lateral groups attached to the *ε*-amine of Lys^7^ (replacing Gly^7^), are compiled in Table S1, Supplementary Materials. The results comprising purity determination, obtained via high-performance liquid chromatography (HPLC) analysis and matrix-assisted laser desorption/ionization–time-of-flight (MALDI-TOF) mass spectrometry (MS), are consistent with the formation of the expected products at a purity >95%.

The labeling of the DT1 reference and the new DT10/11/12 analogs with Tc-99m proceeded at room temperature in alkaline aqueous medium using SnCl_2_ as the reductant in the presence of citrate anions serving as the transfer ligand, as previously detailed [36]. The quality control of radiolabeled products comprising radioanalytical HPLC (single radiopeptide species; Table S2, Supplementary Materials including retention times (*t*_R_) and % purity in two systems) and instant thin-layer chromatography (iTLC, sum of radiochemical impurities [^99m^Tc]TcO_4_^−^, [^99m^Tc]citrate and [^99m^Tc]TcO_2_ × nH_2_O < 2%) verified the formation of the desired radioligands in a radiochemical purity > 98% at molar activities of 20–40 MBq/nmol peptide. Radioligands (Figure 1) were used without further purification in all further experiments; quality controls conducted prior to and after the completion of all biological studies confirmed the preservation of initial radiochemical purity.

### 2.2. In Vitro Studies

#### 2.2.1. Binding Affinities for the Human NTS_1_R

**Figure 2:**
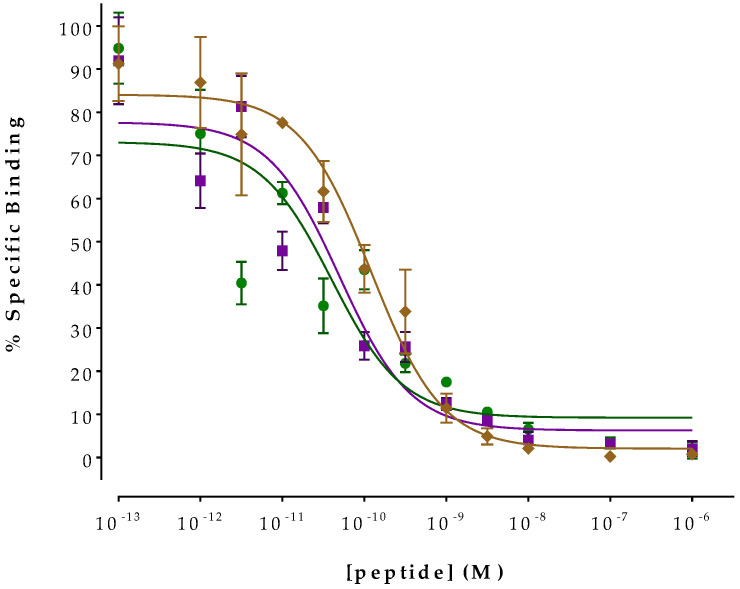
Curves of [^125^I]I-Tyr^3^-NT displacement from NTS_1_R binding sites in WiDr cell membranes according to increasing concentrations of the following: DT10—green line (IC_50_ = 0.06 ± 0.03 nM, n = 3), DT11—violet line (IC_50_ = 0.08 ± 0.05 nM, n = 3) and DT12—brown line (IC_50_ = 0.10 ± 0.03 nM, n = 3); results represent mean IC_50_ values ± sd, n = number of separate experiments in triplicate.

The binding affinities of the new DT1 mimics for the human NTS_1_R were determined via competition binding assays against the [^125^I]I-Tyr^3^-NT radioligand in freshly prepared membrane homogenates of human colon adenocarcinoma WiDr cells [26,47]. The results are summarized in Figure 2, showing that the attachment of any of the lateral groups on Lys^7^ had a negligible impact on the binding affinities of DT10/11/12. The IC_50_s (mean ± standard deviation, sd) determined during the assay were all found in the sub-nanomolar range and were comparable to unmodified DT1 (IC_50_ = 0.14 ± 0.01 nM, n = 4); these were DT10, IC_50_ = 0.06 ± 0.03 nM; DT11, IC_50_ = 0.08 ± 0.05 nM; and DT12, IC_50_ = 0.10 ± 0.03 nM.

#### 2.2.2. Radioligand Internalization in AsPC-1 Cells

**Figure 3:**
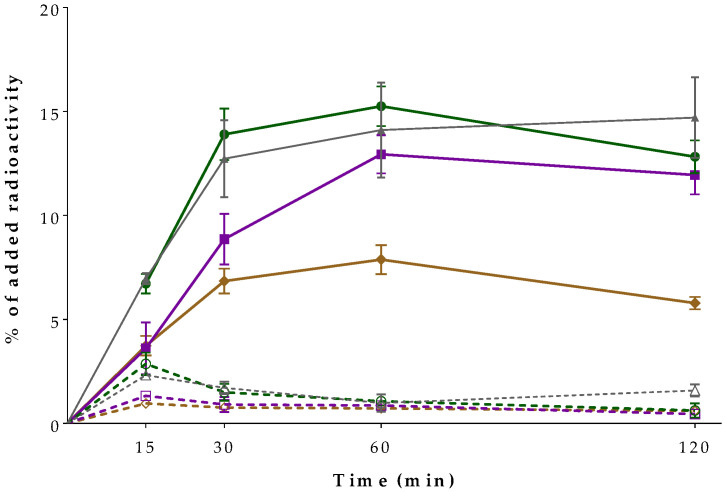
Time-dependent internalization of [^99m^Tc]Tc-DT10 (●, green lines), [^99m^Tc]Tc-DT11 (■, violet lines), [^99m^Tc]Tc-DT12 (♦, brown lines) and [^99m^Tc]Tc-DT1 (reference; ▲, gray lines) in AsPC-1 cells; solid lines correspond to internalized and dotted lines to membrane bound fractions, in all cases. Results represent average values ± sd (n = 3, in triplicate; specific values are included, determined by subtracting non-specifics (found in the presence of 1 μM of NT) from totals).

The comparative time-dependent internalization curves for the new [^99m^Tc]Tc-DT10/11/12 radioligands in human pancreatic adenocarcinoma AsPC-1 cells vs. the [^99m^Tc]Tc-DT1 reference are summarized in Figure 3. The internalization and membrane bound values shown in the diagram are specific ones, calculated by subtracting the respective non-specific values (obtained in the presence of 1 μM of NT) from the totals [26,27]. Comparable internalization rates were displayed by the two ABD-modified analogs, [^99m^Tc]Tc-DT10 and [^99m^Tc]Tc-DT11; these were in the same range as the internalization rates of the parent [^99m^Tc]Tc-DT1. In contrast, [^99m^Tc]Tc-DT12 internalized less compared with all the above radioligands. For example, at 1 h, the specific internalization values per radioligand were as follows: [^99m^Tc]Tc-DT1, 15.1 ± 2.3%; [^99m^Tc]Tc-DT10, 16.3 ± 0.9%; [^99m^Tc]Tc-DT11, 13.4 ± 0.8% (*p* > 0.05 for all comparisons); and [^99m^Tc]Tc-DT12, 8.6 ± 0.7% (*p* < 0.0001 for all comparisons). In all cases, the bulk of radioactivity was found within the cells with a very low portion still bound to the cell membrane, concordant with the profile of receptor–agonist radioligands.

#### 2.2.3. Radioligand Binding to Albumin

**Figure 4:**
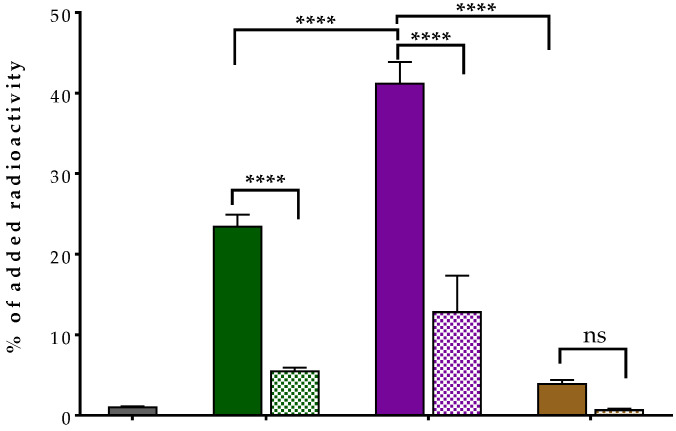
Comparative binding (as shown from left to right) of [^99m^Tc]Tc-DT1 (reference; 1st set of bars—gray), [^99m^Tc]Tc-DT10 (2nd set of bars—green), [^99m^Tc]Tc-DT11 (3rd set of bars—violet) and [^99m^Tc]Tc-DT12 (4th set of bars—brown) to albumin; solid bars correspond to controls and checkered bars to values obtained with the addition of excess ibuprofen. Results represent average values ± sd (n = 3, in triplicate); **** denotes *p* < 0.0001 and ns denotes *p* > 0.05 (not statistically significant).

The ranking of the new radioligands’ capacity to bind to albumin was established in comparison with the [^99m^Tc]Tc-DT1 reference, as summarized in Figure 4. As expected, the ABD-containing analogs, [^99m^Tc]Tc-DT10 and [^99m^Tc]Tc-DT11, displayed considerable albumin binding in this assay. It is interesting to note that [^99m^Tc]Tc-DT11 achieved the highest value (41.2 ± 2.7%), with [^99m^Tc]T6c-DT10 attaining significantly less binding (23.4 ± 1.5%; *p* < 0.0001), a finding showing the favorable effect of the PEG4 spacer on binding to albumin. Another interesting finding is that the addition of ibuprofen [48] to the incubation medium provoked a significant reduction in the binding of both radioligands to albumin (to 12.8 ± 4.5% and 5.5 ± 0.4%, respectively; *p* < 0.0001). Neither [^99m^Tc]Tc-DT12 nor [^99m^Tc]Tc-DT1 displayed measurable levels of albumin binding (<1.0%), a result attributed to their lack of an ABD moiety. 

### 2.3. Animal Experiments

#### 2.3.1. Radioligand Stability in Mice

**Figure 5:**
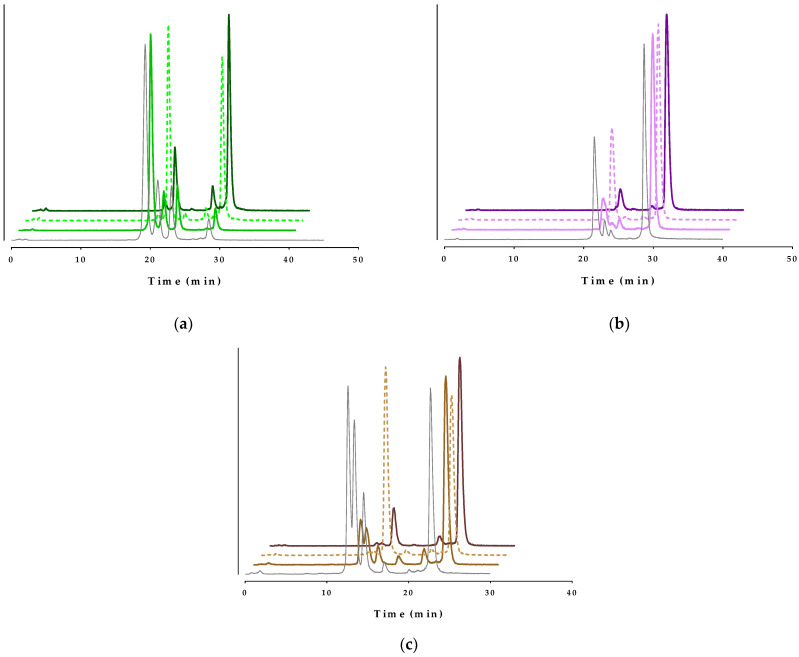
Representative radiochromatograms of the HPLC analysis of mouse blood samples collected 5 min pi of (**a**) [^99m^Tc]Tc-DT10 (green lines), (**b**) [^99m^Tc]Tc-DT11 (violet lines) or (**c**) [^99m^Tc]Tc-DT12 (brown lines), administered consecutively shown from front to back: without treatment (controls, gray solid lines), or treated with Entresto^®^ (lighter-colored solid lines), or with Lis (lighter-colored dotted lines), or with the Entresto^®^ + Lis combination (darker-colored solid lines; HPLC system 2); the percentages of intact radioligand are summarized in Table 1.

The metabolic stability of the new [^99m^Tc]Tc-DT10/11/12 analogs was tested in the peripheral blood of healthy mice at 5 min post-injection (pi). To determine the involvement of neprilysin (NEP) and/or the angiotensin-converting enzyme (ACE) in their in vivo degradation, radioligands were injected in additional groups of mice treated with Entresto^®^, Lis, or their combination. Representative radiochromatograms for each compound and treatment are compiled in Figure 5, while cumulative results are summarized in Table 1, whereby previous data on the stability of [^99m^Tc]Tc-DT1 are included for comparison purposes.

First of all, a significant enhancement in the in vivo stability of all side-chain modified analogs is observed compared with the reference (1.81 ± 0.77% intact). This is especially true for [^99m^Tc]Tc-DT11 (56.56 ± 5.19% intact; *p* < 0.0001) and [^99m^Tc]Tc-DT12 (35.65 ± 5.00% intact; *p* < 0.0001), both carrying the longer lateral chains on Lys^7^ amongst this group of compounds. Another interesting observation is the distinct impact of mice treatment with Entresto^®^ on the stability of these two longer side-chain analogs, namely [^99m^Tc]Tc-DT11 (76.98 ± 3.31% intact; *p* < 0.0001) and [^99m^Tc]Tc-DT12 (58.69 ± 7.17% intact; *p* < 0.0001), vs. the controls, directly implicating NEP as the major catabolizing peptidase. It should be noted that in the case of [^99m^Tc]Tc-DT11, there is no significant difference between the Lis and the control mice groups (*p* > 0.05), or between the Entresto^®^ and the Entresto^®^ + Lis groups (*p* > 0.05), implying the negligible involvement of ACE in its in vivo degradation. On the other hand, both [^99m^Tc]Tc-DT10 and the [^99m^Tc]Tc-DT1 reference require the simultaneous inhibition of both NEP and ACE to reach maximum in vivo stability. Interestingly, [^99m^Tc]Tc-DT12 showed less dependence on ACE inhibition for in vivo stabilization (Lis vs. controls *p* > 0.05 and Entresto^®^ vs. Entresto^®^ + Lis groups *p* < 0.01, Table 1).

#### 2.3.2. Radioligand Biodistribution in AsPC-1 Tumor-Bearing Mice

**Figure 6:**
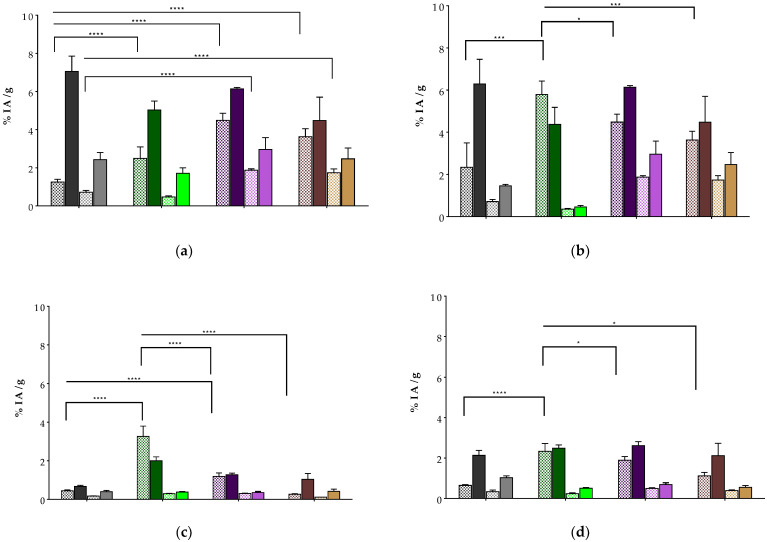
Comparative biodistribution data shown here from left to right for [^99m^Tc]Tc-DT1 (reference; 1st set of bars: gray), [^99m^Tc]Tc-DT10 (2nd set of bars: green), [^99m^Tc]Tc-DT11 (3rd set of bars: violet) and [^99m^Tc]Tc-DT12 (4th set of bars: brown) in SCID mice bearing AsPC-1 xenografts at 4 h (controls—dark checkered bars and Entresto^®^ + Lis-treated mice (only Entresto^®^ treated for [^99m^Tc]Tc-DT11)—dark solid bars) and 24 h pi (controls—light checkered bars and Entresto^®^ + Lis-treated mice (only Entresto^®^ treated for [^99m^Tc]Tc-DT11)—light solid bars) for: (**a**) AsPC-1 tumors; (**b**) kidneys, (**c**) liver and (**d**) intestines; data are expressed as %IA/g and represent average values ± sd, n = 4, and the same scale was kept across tumor and organs for easy comparison purposes; statistically significant differences across radioligands at 4 and 24 h pi, ****: *p* < 0.0001, ***: *p* < 0.001 and *: *p* < 0.05.

The biodistribution of [^99m^Tc]Tc-DT10/11/12 was studied at 4 and 24 h pi in SCID mice bearing AsPC-1 tumors in their flanks. Additional groups of animals were treated with the Entresto^®^ + Lis combination for both time intervals, with the exception of [^99m^Tc]Tc-DT11, with extra groups treated only with Entresto^®^. The specificity of uptake was assessed at 4 h pi using an additional NEP/ACE inhibition animal group receiving an excess NT along with the radioligand for in vivo NTS_1_R blockade. The results, expressed as the percentage of administered activity per gram tumor or tissue (%IA/g) and corresponding to mean values ± sd (n = 4), are summarized in Table 2 ([^99m^Tc]Tc-DT10), Table 3 ([^99m^Tc]Tc-DT11) and Table 4 ([^99m^Tc]Tc-DT12), whereas cumulative data for easy comparison across analogs are selectively presented for AsPC-1 tumors, kidneys, liver and intestines in Figure 6.

All radioligands showed clear uptake in the AsPC-1 tumors in the control groups, which was significantly higher compared with unmodified [^99m^Tc]Tc-DT1 (1.25 ± 0.14% IA/g at 4 h pi), according to the following rank: [^99m^Tc]Tc-DT11 (4.48 ± 0.37% IA/g, *p* < 0.0001), [^99m^Tc]Tc-DT12 (3.63 ± 0.42% IA/g; *p* < 0.0001) and [^99m^Tc]Tc-DT10 (2.49 ± 0.60% IA/g; *p* < 0.0001). The tumor uptake of the latter was significantly enhanced during NEP/ACE inhibition (5.03 ± 0.47% IA/g, *p* < 0.0001), similar to the tumor uptake increase reported for the [^99m^Tc]Tc-DT1 referenc [26,27]. Interestingly, though, the observed tumor enhancement was not statistically significant either in the case of [^99m^Tc]Tc-DT11 during single NEP inhibition (6.14 ± 0.08% IA/g, *p* > 0.05) or of [^99m^Tc]Tc-DT12 during NEP/ACE inhibition (4.48 ± 1.23% IA/g, *p* > 0.05). In all cases, tumor uptake could be significantly reduced in the inhibitor-treated animals receiving excess NT together with the radioligand, demonstrating an NTS_1_R-mediated process. The tumor uptake declined for all analogs at 24 h pi, both in the control and the inhibitor-treated animals, with [^99m^Tc]Tc-DT11 displaying the highest values.

The background radioactivity levels differed across analogs, but consistently declined between 4 h and 24 h pi, both in the control and the inhibitor-treated animal groups. Interestingly, [^99m^Tc]Tc-DT10 displayed the highest radioactivity levels in the control groups in mice kidneys, liver and intestines compared to any other radioligand, including the [^99m^Tc]Tc-DT1 reference, most probably due to the highest levels of radioactivity in the blood (3.46 ± 0.60% IA/g at 4 h pi). On the other hand, [^99m^Tc]Tc-DT11 showed comparable renal values with [^99m^Tc]Tc-DT12 and the [^99m^Tc]Tc-DT1 reference, with lower uptake than [^99m^Tc]Tc-DT10, and this trend remaining consistent in the liver and the intestines. In the inhibitor-treated animals, small increases in the kidneys and intestines, but not in the liver, were observed in the case of [^99m^Tc]Tc-DT11, which overall displayed a very attractive profile for NTS_1_R tumor imaging, either alone or during treatment with the appropriate NEP inhibitor (Figure 6).

#### 2.3.3. SPECT/CT in AsPC-1 Tumor-Bearing Mice

**Figure 7:**
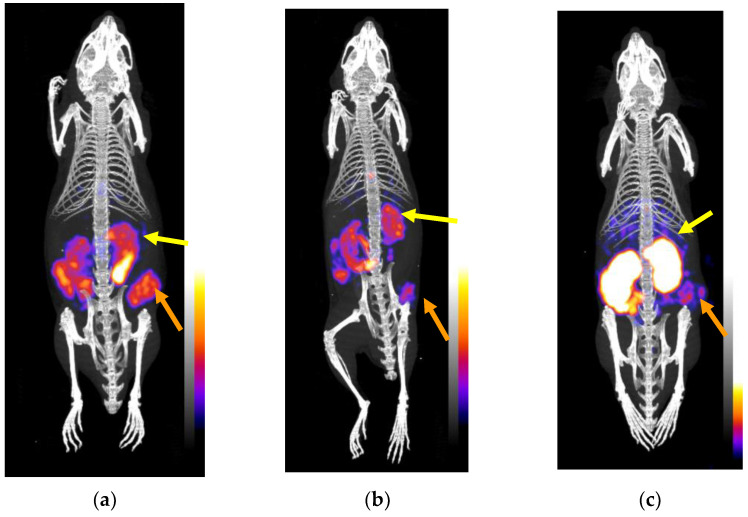
Static whole-body SPECT/CT images of SCID mice bearing AsPC-1 xenografts in their flanks at 4 h pi of (**a**) [^99m^Tc]Tc-DT11 alone or (**b**) together with excess NT for in vivo NTS_1_R blockade, (**c**) with results from [^99m^Tc]Tc-DT1 for comparison purposes; orange arrows are pointing to the human NTS_1_R-expressing xenografts and yellow arrows are directed toward the kidneys. The AsPC-1 tumor was well-delineated by [^99m^Tc]Tc-DT11 in (**a**) but not in (**b**), consistent with an NTS_1_R-mediated process, and the kidneys were visible too. In contrast, rapidly biodegradable [^99m^Tc]Tc-DT1 failed to effectively target the implanted tumor while displaying excessive kidney uptake (**c**). The color bars indicate radioactivity levels (purple being the lowest and white the highest level of accumulation).

The results from the SPECT/CT imaging of mice bearing AsPC-1 tumors in their flanks 4 h after receiving [^99m^Tc]Tc-DT11 alone or excess NT are summarized in Figure 7a and b, respectively. For comparison purposes, a third animal that instead received [^99m^Tc]Tc-DT1 is included in the Figure 7c. The implanted tumor is excellently delineated by [^99m^Tc]Tc-DT11 in the control, but not in the NT-treated mouse, in support of an NTS_1_R-mediated uptake. The kidneys are also clearly visible in both cases. In contrast, [^99m^Tc]Tc-DT1 failed to clearly visualize the implanted tumor displaying excessive kidney accumulation. These findings corroborated biodistribution results.

## 3. Discussion

The propensity of NT and NT (8–13) radioligands for rapid degradation in the biological milieu has been a major limiting factor for their use as radiotheranostic tools against NTS_1_R-expressing human tumors [19,20,21]. Several attempts to improve their metabolic stability were originally directed at changes of peptide bonds, amino acid replacements, or C-terminus modifications, most often occurring at the cost of internalization efficacy, receptor affinity, or tumor targeting and pharmacokinetics [10,11,14,15,16,17,18]. Unluckily, most studies to determine metabolic stability have involved the in vitro incubation of test radiopeptides in plasma or serum, a process disregarding the impact of ectoenzymes [16,49]. The latter, anchored onto the epithelial cells of vasculature and major organs of the body, break-down circulating radioligands on the way to the target, and eventually compromising tumor uptake. Based on previous studies on the metabolic fate of NT and its analogs in the body [22,23,24,25], we could confirm the prominent and synergetic role of ACE and NEP in the rapid degradation of [^99m^Tc]Tc-DT1 when entering the circulation [26,27]. Most importantly, we were able to show that the in situ inhibition of ACE/NEP exerted a strong stabilization effect on [^99m^Tc]Tc-DT1 and its analogs in peripheral blood, which translated into a markedly enhanced uptake in NTS_1_R-positive tumors in mice [26,27]. Even though improved diagnostic efficacy during single NEP inhibition has been recently confirmed for a biodegradable radiolabeled gastrin analog in medullary thyroid carcinoma patients [30,31,32], the clinical translation of this concept in the case of NT radioligands would require dual ACE/NEP inhibition. Understandably, this is a more complex and challenging task with regards to ethical committee approvals, biosafety and the development/optimization of clinical protocols. Amongst the methods used to achieve metabolic stability of [^99m^Tc]Tc-DT1 mimics without negatively affecting other important biological responses, we recently attempted palmitoylation on Lys^7^ in [^99m^Tc]Tc-DT9. Interestingly, [^99m^Tc]Tc-DT9 showed excellent properties in all in vitro assays, full ACE/NEP resistance and high uptake in xenografted AsPC-1 tumors in mice, but the pharmacokinetics required improvement [36].

To overcome this handicap, we proceeded next to replace the Lys^7^-attached lipophilic palmitoyl chain using either ABD groups of different lengths ([^99m^Tc]Tc-DT10/11) or a hydrophilic arm of a similar length, not binding to albumin ([^99m^Tc]Tc-DT12) (Figure 1). These interventions turned out to be well tolerated by the NTS_1_R, as verified via competition binding assays against the [^125^I]I-Tyr^3^-NT radioligand (Figure 2). The sub-nanomolar IC_50_ values determined for DT10/11/12 were comparable to that of DT1, further corroborating the suitability of position 7 in the NT sequence for coupling pendant chains [36,41,42,43]. Likewise, the internalization capabilities of the respective ABD-modified [^99m^Tc]Tc-DT10/11 radioligands in AsPC-1 cells were comparable with [^99m^Tc]Tc-DT1, albeit lower for the hydrophilic PEG6-carrying [^99m^Tc]Tc-DT12 (Figure 3). As expected, both [^99m^Tc]Tc-DT12 and the [^99m^Tc]Tc-DT1 reference lacking an ABD arm failed to bind to albumin (Figure 4). In contrast, significant binding to albumin was evident in the case of the two ABD-carrying radioligands, but its extent differed. The PEG4-containg [^99m^Tc]Tc-DT11 bound significantly more strongly to albumin compared with [^99m^Tc]Tc-DT10, revealing the role of steric factors in this interaction.

The interplay of albumin binding and/or steric factors was more apparent in the metabolic stability variations across analogs revealed through the comparative analysis of blood from mice receiving the radioligands without or during NEP/ACE inhibition (Figure 5; Table 1). Accordingly, the short-chain albumin-binding [^99m^Tc]Tc-DT10 turned out to be degraded much faster in control mice than the longer-chain [^99m^Tc]Tc-DT11 and [^99m^Tc]Tc-DT12. The latter, although not equally ACE/NEP-resistant to palmitoylated [^99m^Tc]Tc-DT9, demonstrated significantly higher stability than [^99m^Tc]Tc-DT10. Treatment with Entresto^®^/Lis led to even further stability improvements vs. the controls. It should be noted that in the case of [^99m^Tc]Tc-DT11, the single use of Entresto^®^ sufficed for full stabilization, a finding consistent with ACE resistance. On the other hand, [^99m^Tc]Tc-DT12, carrying a chain not binding to albumin and of similar length to palmitoyl, failed to achieve the same stability levels with [^99m^Tc]Tc-DT11, either in controls or during single NEP inhibition (*p* < 0.001; Table 1). This finding implies the synergetic steric and albumin-binding influences of the lateral Lys^7^ chain on metabolic stability. 

The tumor-targeting capability and overall biodistribution of the three analogs was compared in mice bearing subcutaneous AsPC-1 tumors (Table 2, Table 3 and Table 4; Figure 6), allowing for a few important conclusions to be drawn. Firstly, all radioligands achieved higher uptake in the implanted tumors vs. unmodified [^99m^Tc]Tc-DT1, with longer-chain [^99m^Tc]Tc-DT11 and [^99m^Tc]Tc-DT12 reaching the highest values, in line with their enhanced metabolic stability. In particular, [^99m^Tc]Tc-DT11, combining the highest stability in this group of compounds with a retained internalization capacity in AsPC-1 cells, yielded the highest tumor values. Secondly, [^99m^Tc]Tc-DT11 ranked first in tumor uptake during single NEP inhibition compared to all other radioligands during parallel ACE/NEP inhibition. Notably, the enhancement of tumor uptake during peptidase inhibition was much more pronounced in the case of the fast biodegradable [^99m^Tc]Tc-DT1 and [^99m^Tc]Tc-DT10, but lower and not statistically significant for [^99m^Tc]Tc-DT11. Thirdly, all side-chain modified radioligands showed significantly lower background radioactivity levels compared with palmitoylated [^99m^Tc]Tc-DT9 [36]. For example, it is interesting to compare the highest uptake in the controls across these analogs, shown by [^99m^Tc]Tc-DT10 with [^99m^Tc]Tc-DT9 in the kidneys (5.79 ± 0.63% IA/g vs. 9.53 ± 1.18% IA/g at 4 h pi; 0.36 ± 0.03% IA/g vs. 3.81 ± 0.54% IA/g 24 h pi) and the liver (3.27 ± 0.53% IA/g vs. 29.56 ± 3.77% IA/g at 4 h pi; 0.29 ± 0.02% IA/g vs. 12.93 ± 1.66% IA/g at 24 h pi). In addition, the tumor uptake of [^99m^Tc]Tc-DT9 was comparable to that of [^99m^Tc]Tc-DT11 (*p* > 0.05), but could be only partially attributed to an NTS_1_R-mediated process due to high radioactivity levels in the blood and only partial reduction during the in vivo blockade of the receptor.

In view of the above, it is rational to conclude that the substitution of palmitoyl in [^99m^Tc]Tc-DT9 by more hydrophilic lateral chains led to pharmacokinetic improvements in newly introduced analogs. Albumin binding and steric factors synergistically operated in [^99m^Tc]Tc-DT11, which, combining high NTS_1_R affinity, internalization capacity, metabolic stability, tumor targeting and improved pharmacokinetics, outperformed unmodified [^99m^Tc]Tc-DT1 and its mimics. These conclusions were further supported by the comparison of [^99m^Tc]Tc-DT11 and [^99m^Tc]Tc-DT1 on SPECT/CT (Figure 6), qualifying [^99m^Tc]Tc-DT11 for further evaluation in patients with NTS_1_R-positive tumors.

## 4. Materials and Methods

### 4.1. Chemicals and Radioligands

#### 4.1.1. Chemicals and Radionuclides

The chemicals used were reagent grade and were obtained from common commercial sources, whereas the HPLC solvents were HPLC grade. NT was purchased from Bachem (Bubendorf, Switzerland). Entresto^®^ (Novartis AG, Basel, Switzerland) was bought from a local pharmacy, while Lis was obtained from Sigma-Aldrich (St. Louis, MI, USA). The peptide conjugates DT1, DT10, DT11 and DT12 were provided by PiChem Forschungs und Entwicklungs GmbH (Raaba-Grambach, Austria). The analogs were dissolved at 2 mg/mL in doubly distilled H_2_O, equally distributed in 50 μL aliquots in Eppendorf Protein LoBind tubes and stored at −20 °C.

For labeling the peptide conjugates with Tc-99m, a commercial [^99^Mo]Mo/[^99m^Tc]Tc generator (Ultra-Technekow V4 Generator, Curium Pharma, Petten, The Netherlands) was eluted, affording [^99m^Tc]NaTcO_4_ in normal saline. [^125^I]NaI in dilute sodium hydroxide solution (pH 8–11) was obtained from Perkin Elmer (Waltham, MA, USA) for the preparation of [^125^I]I-Tyr^3^-NT, used in the competition binding assays.

#### 4.1.2. Radioligands—Radiochemistry

For labeling of DT10/11/12 and of the DT1 reference, [^99m^Tc]NaTcO_4_ (420 μL of generator eluate) was added in a LoBind Eppendorf tube containing phosphate buffer (0.5 M, pH 11.5, 50 μL); the following solutions were then consecutively added: sodium citrate (0.1 M, 5 μL); stock solution of the peptide conjugate (15 μL, 15 nmol); and SnCl_2_ freshly dissolved in EtOH (10 μL, 10 μg). After a 30 min incubation at room temperature (RT), the pH of the labeling reaction mixture was adjusted to 7.4 with the addition of 0.1 M HCl.

Quality control comprised the HPLC and iTLC methods. The HPLC analyses were conducted on a Waters Chromatograph equipped with binary detection modes, comprising a 2998 photodiode array UV detector (Waters, Vienna, Austria) and a Gabi gamma detector (Raytest RSM Analytische Instrumente GmbH, Straubenhardt, Germany), and monitored via the Empower Software 3 (Waters, Milford, MA, USA). A Symmetry Shield RP-18 (5 μm, 3.9 mm × 20 mm) cartridge column (Waters, Eschborn, Germany) was eluted at a flow rate of 1 mL/min with a linear gradient (system 1): from 100% A/0% B to 60% A/40% B in 20 min (A: 0.1% aqueous TFA and B: MeCN). Whatman 3 mm chromatography paper strips (GE Healthcare, Chicago, IL, USA) were used for iTLC analyses. Strips were developed up to 10 cm from the application site with either 5 M NH_4_AcO/MeOH 1:1 (*v*/*v*) (detection of any reduced, hydrolyzed technetium [^99m^Tc]TcO_2_ × nH_2_O at R_f_ = 0) or acetone (detection of free unreduced [^99m^Tc]TcO_4−_ at R_f_ 1). A γ-counter (automated multi-sample well-type instrument with a NaI(Tl) 3’´ crystal, Canberra Packard Cobra^TM^ Quantum U5003/1, Auto-Gamma^®^ counting system; Canberra Packard, Ramsey, MN, USA) was used for the sample radioactivity measurements. For all subsequent tests, a radioligand solution was prepared in the desired radioactivity concentration in phosphate-buffered saline (PBS, pH 7.4)/EtOH *v*/*v* 9/1; aliquots thereof were tested before and after the completion of all experiments.

The labeling of NT with I-125 was achieved following the chloramine-T method and the resulting [^125^I]I-Tyr^3^-NT radioligand was purified via HPLC, as previously described [36]. The HPLC-isolated [^125^I]I-Tyr^3^-NT was diluted in 0.1% BSA-PBS buffer at a molar activity of 74 GBq/μmol; the solution was equally distributed in LoBind Eppendorf tubes and stored at −20 °C until use in the competition binding assays.

All manipulations with radioactivity solutions and samples were performed by authorized personnel, in dedicated and licensed laboratories, abiding by European radiation-safety guidelines and supervised by the Greek Atomic Energy Commission (license # A/435/17092/2019).

### 4.2. Cell Studies

#### 4.2.1. Cell Lines—Cell Culture

Colorectal adenocarcinoma WiDr cells (LGC Promochem; Teddington, UK) were grown in McCoy’s GLUTAMAX-I medium supplemented with 10% (*v*/*v*) fetal bovine serum (FBS), 100 U/mL of penicillin and 100 µg/mL of streptomycin. Pancreatic adenocarcinoma AsPC-1 cell lines (LGC Standards GmbH; Wesel, Germany) were cultured in GLUTAMAX-I Roswell Park Memorial Institute-1640 (RPMI), also supplemented with 10% (*v*/*v*) fetal bovine serum (FBS), 100 U/mL of penicillin and 100 µg/mL of streptomycin. Cells were maintained in 75 cm^2^ flasks at 37 °C (95% humidity, 5% CO_2_) in a Heal Force SMART CELL HF-90 incubator (Shanghai, China). Weekly splittings were performed at a ratio of 1:3–1:5 when 80–90% confluency was reached by treating the cells with a Trypsin/EDTA (0.05%/0.02% *w*/*v*) solution. Culture media were obtained from Thermo Fisher Scientific LSG (Waltham, MA, USA), while supplements and the Trypsin/EDTA solution were purchased from Biochrom KG Seromed (Berlin, Germany).

#### 4.2.2. Competition Binding Assays

A solution of the [^125^I]I-Tyr^3^-NT radioligand in cold binding buffer (BB: 50 mM of HEPES, 5.5 mM of MgCl_2_, 0.1 mg/mL bacitracin, 1% *w*/*v* BSA, pH 7.4; 70 µL, 40,000 dpm, 50 pM end-concentration per assay-tube) and a test peptide dilution series ranging from 10^−12^ to 10^−5^ M in cold BB were prepared and placed on ice. Aliquots of the cell membrane homogenates from WiDr cells (collected and stored at −80 °C in Tris/EDTA:10 mM of Tris, 0.1 mM of EDTA, pH 7.4) were thawed, combined and diluted in cold BB. In RIA tubes in triplicate, the test peptide (30 µL) of each concentration point, radioligand (70 µL) and membrane homogenate (200 µL) were consecutively added and thoroughly vortexed. Samples were placed for 1 h in the Incubator-Orbital Shaker (MPM Instr. Srl; Bernareggio, Italy) at 22 °C under constant steering. The binding reaction was terminated by placing the samples on ice, followed by the addition of ice-cold washing buffer (10 mM of HEPES, 150 mM of NaCl, pH 7.4) on a 48-sample Brandel Cell Harvester (Adi Hassel Ingenieur Büro, Munich, Germany) loaded with Whatman GF/B filters presoaked for 1 h in BB. The samples were aspirated, and individual filters corresponding to each tube were collected in separate plastic tubes and measured for radioactivity on the γ-counter. Inhibition curves were plotted and the half maximal inhibitory concentration (IC_50_) values were calculated by applying a nonlinear one-site model (GraphPad Prism—6 Software, San Diego, CA, USA). The results for each compound are given as the average IC_50_ values ± sd of at least three independent repetitions of the experiment performed in triplicate.

#### 4.2.3. Cell Internalization Experiments 

The time-dependent internalization of [^99m^Tc]Tc-DT10/11/12 was directly compared with [^99m^Tc]Tc-DT1 via incubation in AsPC-1 cells at 37 °C for 15 min, 30 min, 1 h and 2 h. The cells were seeded in 6-well plates (1 × 10^6^ per well) and left in the incubator overnight. The next day, the cells were washed twice with ice-cold internalization medium (IM, RPMI supplemented with 1% FBS). The plates were placed on the bench and the following solutions were successively added per well: warm IM (1200 µL at 37 °C), radioligand (250 fmol in 150 µL of IM) and either IM (150 µL; 3 upper wells: total series) or NT (10^−5^ M in IM, 3 bottom wells: non-specific series). The plates were placed in the incubator at 37 °C and, at the predetermined time points, were retrieved and placed on ice. The supernatants were collected in separate RIA tubes and the cells were washed with ice-cold phosphate-buffered saline (pH 7.4) supplemented with 0.5% *w*/*v* BSA (IPBS; 1 mL); washings were collected and combined with the respective supernatants. The cells were next treated with acid glycine buffer (AGB, 50 mM of glycine, 0.1 M NaCl, pH 2.8; 2 × 5 min with 600 µL) and the supernatants corresponding to the membrane bound fraction (MB) were collected. After an additional wash with ice-cold IPBS (1 mL), the cells were lysed with 1 M NaOH (2 × 600 µL); the lysates were combined and collected (internalized fraction). The three fractions collected per well in separate RIA tubes (supernatant, MB and internalized) were measured for their radioactivity content on the γ-counter. The radioactivity of each fraction was then assessed as a percentage of the initial activity added per well. By subtracting the non-specific fractions from the respective total fractions, the specific MB and internalization fractions could be determined. Each assay was performed at least three times in triplicate. The results were expressed as mean ± sd.

#### 4.2.4. Albumin Binding Assays 

The binding of the new [^99m^Tc]Tc-DT10/11/12 and the [^99m^Tc]Tc-DT1 reference to albumin was compared via the incubation of radioligands in BSA-treated 6-well plates [50,51,52]. Accordingly, HEPES-buffered saline (150 mM of NaCl, 20 mM of HEPES, pH 7.4) supplemented with 0.5% *w*/*v* BSA (1200 µL of HBS-BSA) was added to each well, followed by the radioligand (250 fmol peptide, in 150 µL of HBS) and either HBS-BSA (150 µL, total binding) or Ibuprofen (22.2 mg/mL in HBS, 150 µL), to reach a total final volume of 1.5 mL. The plates were incubated in triplicate at 37 °C for 30 min in the incubator. Next, the supernatants were discarded and the wells were treated with 1 M NaOH (2 × 600 µL). The lysates were combined and collected in RIA tubes for radioactivity measurement on the γ-counter. Binding to albumin was calculated as a percentage of the measured activity vs. the activity originally added per well. The experiments were performed at least three times in triplicate and the results represent the average binding to albumin ± sd.

### 4.3. Animal Studies

#### 4.3.1. Healthy and Tumor-Bearing Immunosuppressed Mice 

Healthy Swiss Albino mice (36 animals, 8–10 weeks of age, body weight: 30 ± 5 g) and male mice with severe combined immunodeficiency (SCID) (63 animals, 23.1 ± 1.6 g body weight, six weeks of age on arrival day) were purchased from NCSR “Demokritos” Animal House (Athens, Greece). Animals were housed in licensed facilities (EL 25 BIO exp021) under sterile conditions with 12 h day/night cycles and were provided with sterilized chow food and drinking water ad libitum; they were used in all further experiments after an acclimatization period.

For tumor induction, the SCID mice were subcutaneously injected in the right flank with a sterile suspension of freshly harvested AsPC-1 cells (5 × 10^6^ cells in 150 µL of sterile PBS) under aseptic conditions. After 3–4 weeks, well-palpable masses developed at the implantation sites (≈100 µg tumor mass) and the animals were randomly divided into suitable groups for biodistribution (60 mice) or SPECT/CT imaging (3 mice).

#### 4.3.2. Metabolic Studies in Healthy Mice

The healthy Swiss Albino mice were randomly divided into groups of three and injected via their tail vein with the radioligand (100 μL, 3 nmol in vehicle: PBS/EtOH 9/1 *v*/*v*) plus the following: (i) vehicle (100 μL)—controls; (ii) Lis (100 μg in 100 μL vehicle) [26,27,29,36]—Lis; (iii) vehicle (100 μL) 30 min after receiving per os Entresto^®^ (a suspension of 12 mg/200 μL in H_2_O per animal)—Entresto^®^; and (iv) plus Lis (100 μg in 100 μL vehicle) 30 min after receiving per os Entresto^®^—Entresto^®^ + Lis. Individual Entresto^®^ doses (12 mg/200 μL per animal) were prepared by grinding Entresto^®^ pills (200 mg, corresponding to 24 mg/26 mg of sacubitril/valsartan per pill; Novartis AG, Basel, Switzerland) into a fine powder in a mortar, and dividing and suspending it in tap water to form a slurry [26,27,33,34,35,36]. Animals were euthanized 5 min pi and their blood was collected using a prechilled penicillin heparinized syringe in ice-cold LoBind Eppendorf vials containing 0.1 mM of EDTA solution (20 µL).

The collected blood samples were centrifuged for 10 min at 5000 rpm at 4 °C; the plasma was collected in new tubes and diluted with an equal volume of MeCN. After a brief mixing, the samples were centrifuged at 15,000 rpm for 10 min at 4 °C (in a Hettich Universal 320 R centrifuge; Tuttlingen, Germany). The supernatants were transferred into glass vials and concentrated under a gentle nitrogen gas stream and mild warming. The concentrates (≈100 µL) were diluted in physiological saline (300–450 µL) and passed through a Millex GV filter (0.22 μm, 13 mm diameter, Millipore, Milford, CT, USA). The sample radioactivity was measured after each step of the process in the dose calibrator (CURIEMENTOR 4, PTW Freiburg-GmbH; Freiburg, Germany) to alert us to losses to containers due to sticking. Aliquots of the filtrate were analyzed via HPLC (equipment and cartridge column used as in Section 4.1.2.) by applying the following gradient at a 1 mL/min flow rate (system 2): from 100% A/0% B to 60% A/40% B in 40 min (A: 0.1% aqueous TFA and B: MeCN). The peak corresponding to the intact radiotracer was determined via the co-injection of aliquots of the labeling solution, yielding identical retention times (*t*_R_). Results were obtained from three mice per analog per treatment and were calculated as the average percentage of intact radioligand ± sd.

#### 4.3.3. Biodistribution in Tumor-Bearing SCID Mice

Biodistribution was conducted in SCID mice bearing subcutaneous AsPC-1 tumors in their flanks (see Section 4.3.1.). On the day of the experiment, the animals were randomly divided into groups of four and injected through the tail vein with a bolus of the radioligand (100 μL, 3 pmol in vehicle: PBS/EtOH 9/1 *v*/*v*) plus the following: (i) vehicle (100 μL; controls at 4 and 24 h pi), or (ii) Lis (100 μg in 100 μL vehicle) after having received 30 min Entresto^®^ per os (200 μL, 12 mg; Entresto^®^ + Lis groups at 4 and 24 h pi); and (iii) excess NT and Lis (100 μg of NT and 100 μg of Lis in 100 μL of vehicle—NTS_1_R block) following treatment with Entresto^®^ (200 μL, 12 mg) 30 min in advance. In the case of [^99m^Tc]Tc-DT11, Lis was not administered in any of the mice groups (controls, Entresto^®^, and block + Entresto^®^). At the predetermined time intervals, the mice were euthanized, weighed and their blood, tissue samples of choice and tumors were collected and weighed. The sample radioactivity was measured on the γ-counter together with proper standards of the injected dose. The results were calculated as a percentage of injected activity per gram tissue (%IA/g) and provided as mean %IA/g values ± sd. For comparisons, a two-way ANOVA with Tukey’s post hoc analysis was applied (PRISM^TM^ GraphPad—6 Software, San Diego, CA, USA). *p* values < 0.05 were considered statistically significant.

#### 4.3.4. SPECT/CT Imaging of Tumor-Bearing SCID Mice

Static SPECT/CT images were obtained in three SCID mice bearing subcutaneous AsPC-1 tumors in their flanks 4 h after the injection of [^99m^Tc]Tc-DT11 alone (100 μL, ≈50 MBq, 1.5 nmol total peptide in vehicle (see Section 4.3.3.)—control), or together with excess NT (block), while a third mousee instead received [^99m^Tc]Tc-DT1 (100 μL, ≈50 MBq, 1.5 nmol total peptide in vehicle) for direct comparison purposes. The mice were euthanized at 4 h pi and tomographic SPECT/CT imaging was performed using the y-CUBE/x-CUBE systems (Molecubes, Ghent, Belgium) and processed, as previously described [26].

The mice experiments complied with European and national regulations and the study protocols were approved by the Department of Agriculture and Veterinary Service of the Prefecture of Athens (#440448, 01-06-2021 for the stability studies and #440451, 01-06-2021 for the biodistribution and imaging studies).

## 5. Conclusions

The present study has further confirmed the suitability of position 7 in NT-based radioligands, such as [^99m^Tc]Tc-DT1, for attaching various types of pendant chains in order to enhance the in vivo metabolic stability. Both steric factors (lateral chain length) and binding to albumin (ABD-coupled residues) were found to influence the resistance of the resulting radioligands to the major involved peptidases, ACE and NEP. At the same time, lateral chains affected other biological features of new analogs, such as the internalization rates, tumor uptake and pharmacokinetics. Amongst the new analogs studied herein, [^99m^Tc]Tc-DT11, whereby an ABD-group was tethered to Lys^7^ via a hydrophilic PEG4 linker, combines improved in vivo stability with fast cell internalization and the highest tumor uptake in mice, not only during single NEP inhibition regimens [26,27] but also when injected alone. Compared with either unmodified [^99m^Tc]Tc-DT1 or Lys^7^-palmitoylated [^99m^Tc]Tc-DT9 [36], [^99m^Tc]Tc-DT11 achieved a markedly superior biological profile, qualifying for evaluation in patients applying SPECT/CT. It should be noted that the recent technological developments in SPECT instrumentation [53,54,55] and the wide availability of high-quality Tc-99m in hospitals [56,57,58] render the prospects of [^99m^Tc]Tc-DT11, as a useful diagnostic candidate for NTS_1_R-expressing human tumors, quite attractive.

## Figures and Tables

**Table 1:** Stabilities of [^99m^Tc]Tc-DT1 (reference), [^99m^Tc]Tc-DT10, [^99m^Tc]Tc-DT11 and [^99m^Tc]Tc-DT12 in peripheral mouse blood 5 min pi without treatment (controls), or treated with Entresto^®^, or Lis, or their combination (Entresto^®^ + Lis).

	[^99m^Tc]Tc-DT1 ^1^	[^99m^Tc]Tc-DT10	[^99m^Tc]Tc-DT11	[^99m^Tc]Tc-DT12
Control	1.81 ± 0.77 (n = 4)	5.09 ± 0.89 (3)	56.56 ± 5.19 (3)	35.65 ± 5.00 (3)
Entresto^®^	5.46 ± 3.86 (n = 5)	5.15 ± 1.58 (3)	76.98 ± 3.31 (3)	58.69 ± 7.17 (3)
Lis	18.77 ± 2.54 (n = 3)	37.14 ±6.49 (3)	66.89 ± 1.24 (3)	42.63 ± 2.79 (3)
Entresto^®^ + Lis	63.80 ± 7.51 (n = 3)	68.02 ± 3.01 (3)	86.33 ± 1.94 (3)	73.98 ± 4.93 (3)

^1^ Metabolic stability results for [^99m^Tc]Tc-DT1 have been adapted from [26,27]; data represent the mean percentage of intact radioligand ± sd; number of experiments are shown in parentheses.

**Table 2:** Biodistribution of [^99m^Tc]Tc-DT10 in SCID mice bearing AsPC-1 xenografts at 4 h (block, controls and Entresto^®^ + Lis treated) and 24 h pi (controls and Entresto^®^ + Lis treated); data are expressed as %IA/g and represent average values ± sd, n = 4.

Organs/Tissues	[^99m^Tc]Tc-DT10 (%IA/g)				
	4 h			24 h	
	Block	Controls	Entresto^®^ + Lis	Controls	Entresto^®^ + Lis
Blood	0.82 ± 0.25	3.46 ± 0.60	1.20 ± 0.26	0.04 ± 0.01	0.05 ± 0.01
Liver	1.77 ± 0.21	3.27 ± 0.53	2.00 ± 0.20	0.29 ± 0.02	0.38 ± 0.04
Heart	0.28 ± 0.06	1.12 ± 0.31	0.43 ±0.07	0.04 ± 0.01	0.04 ± 0.01
Kidneys	3.93 ± 1.25	5.79 ± 0.63	4.38 ± 0.81	0.36 ± 0.03	0.45 ± 0.07
Stomach	0.43 ± 0.12	1.03 ± 0.29	0.90 ± 0.34	0.17 ± 0.04	0.11 ± 0.03
Intestines	1.04 ± 0.10	2.34 ± 0.39	2.49 ± 0.16	0.24 ± 0.04	0.51 ± 0.04
Spleen	0.66 ± 0.08	0.65 ± 0.11	1.10 ± 0.22	0.10 ± 0.02	0.26 ± 0.03
Muscle	0.13 ± 0.04	0.37 ±0.05	0.16 ± 0.04	0.01 ± 0.01	0.02 ± 0.01
Lungs	0.84 ± 0.17	2.15 ± 0.25	1.30 ± 0.48	0.16 ± 0.03	0.18 ± 0.04
Pancreas	0.20 ± 0.03	0.51 ± 0.11	0.30 ± 0.16	0.02 ± 0.00	0.03 ± 0.01
AsPC-1 Tumor	1.60 ± 0.67	2.49 ± 0.60	5.03 ± 0.47	0.47 ± 0.06	1.71 ± 0.28

**Table 3:** Biodistribution of [^99m^Tc]Tc-DT11 in SCID mice bearing AsPC-1 xenografts at 4 h (block, controls and Entresto^®^ treated) and 24 h pi (controls and Entresto^®^ treated); data are expressed as %IA/g and represent average values ± sd, n = 4.

Organs/Tissues	[^99m^Tc]Tc-DT11 (%IA/g)				
	4 h			24 h	
	Block	Controls	Entresto^®^	Controls	Entresto^®^
Blood	0.08 ± 0.29	0.99 ± 0.35	0.93 ± 0.12	0.07 ± 0.01	0.08 ± 0.01
Liver	0.31 ± 0.02	1.19 ± 0.18	1.27 ± 0.08	0.31 ± 0.02	0.35 ± 0.05
Heart	0.04 ± 0.01	0.39 ± 0.10	0.34 ± 0.06	0.04 ± 0.01	0.08 ± 0.02
Kidneys	0.62 ± 0.06	3.11 ± 0.32	3.57 ± 0.36	0.62 ± 0.06	0.80 ± 0.05
Stomach	0.19 ± 0.02	0.71 ± 0.09	0.63 ± 0.13	0.19 ± 0.02	0.16 ± 0.04
Intestines	0.50 ± 0.04	1.89 ± 0.18	2.61 ± 0.20	0.50 ± 0.04	0.69 ± 0.09
Spleen	0.44 ± 0.14	0.85 ± 0.06	1.37 ± 0.18	0.44 ± 0.14	0.60 ± 0.09
Muscle	0.03 ± 0.01	0.14 ± 0.04	0.17 ± 0.01	0.03 ± 0.01	0.04 ± 0.00
Lungs	0.18 ± 0.03	0.86 ± 0.21	1.23 ± 0.15	0.18 ± 0.03	0.34 ± 0.06
Pancreas	0.07 ± 0.02	0.25 ± 0.04	0.33 ± 0.06	0.07 ± 0.02	0.08 ± 0.02
AsPC-1 Tumor	1.87 ± 0.07	4.48 ± 0.37	6.14 ± 0.08	1.87 ± 0.07	2.96 ± 0.63

**Table 4:** Biodistribution of [^99m^Tc]Tc-DT12 in SCID mice bearing AsPC-1 xenografts at 4 h (block, controls and Entresto^®^ + Lis treated) and 24 h pi (controls and Entresto^®^ + Lis treated); data are expressed as %IA/g and represent average values ± sd, n = 4.

Organs/Tissues	[^99m^Tc]Tc-DT12 (%IA/g)				
	4 h			24 h	
	Block	Controls	Entresto^®^ + Lis	Controls	Entresto^®^ + Lis
Blood	0.06 ± 0.02	0.05 ± 0.01	0.06 ± 0.01	0.05 ± 0.01	0.07 ± 0.02
Liver	0.79 ± 0.38	0.26 ± 0.04	1.04 ± 0.31	0.11 ± 0.00	0.41 ± 0.11
Heart	0.05 ± 0.02	0.05 ± 0.01	0.09 ± 0.02	0.05 ± 0.04	0.06 ± 0.02
Kidneys	3.11 ± 1.29	2.43 ± 0.51	6.92 ± 2.55	0.68 ± 0.10	0.83 ± 0.20
Stomach	0.28 ± 0.17	0.19 ± 0.05	0.36 ± 0.06	0.08 ± 0.01	0.14 ± 0.07
Intestines	1.41 ± 1.04	1.12 ± 0.17	2.12 ± 0.62	0.40 ± 0.03	0.55 ± 0.09
Spleen	1.01 ± 0.68	0.28 ± 0.02	2.23 ± 0.81	0.20 ± 0.03	0.97 ± 0.30
Muscle	0.02 ± 0.02	0.03 ± 0.00	0.06 ± 0.02	0.03 ± 0.01	0.04 ± 0.01
Lungs	0.35 ± 0.12	0.12 ± 0.00	0.65 ± 0.15	0.08 ± 0.01	0.26 ± 0.02
Pancreas	0.08 ± 0.04	0.06 ± 0.00	0.12 ± 0.02	0.05 ± 0.01	0.06 ± 0.01
AsPC-1 Tumor	0.56 ± 0.18	3.63 ± 0.42	4.48 ± 1.23	1.74 ± 0.20	2.47 ± 0.57

## Data Availability

Data are contained within the article or Supplementary Material.

## Supplementary Materials

The following supporting information can be downloaded at: https://www.mdpi.com/article/10.3390/ijms242115541/s1.
