# Supplementary material for: Side-Chain Modified [99mTc]Tc-DT1 Mimics: A Comparative Study in NTS1R-Positive Models
Source: Int J Mol Sci. 2023 Oct 24;24(21):15541. doi: 10.3390/ijms242115541 (PMC10647616; doi:10.3390/ijms242115541)
Supplement: Supplementary file 1 [file ijms-24-15541-s001.zip › ijms-2677270-supplementary.pdf]

International Journal of Molecular Sciences

Article

Supplementary Materials: Side-chain modified [^{99m}Tc]Tc-DT1 mimics: A comparative study in NTS$_1$R-positive models

Panagiotis Kanellopoulos [1], Berthold A. Nock [1], Maritina Rouchota [2], George Loudos [2], Eric P. Krenning [3] and Theodosia Maina [1,*]

Analytical data for DT10, DT11 and DT12

Analytical data for the DT1 (N$_4$-Gly^7-Arg-Arg-Pro-Tyr-Ile-Leu-OH; N$_4$, 6-(carboxy)-1,4,8,11-tetraazaundecane) mimics DT10 ([(MPBA)Lys^7]DT1; MPBA, (4-(4-methylphenyl)butyric acid), DT11 ([(MPBA-PEG4)Lys^7]DT1; PEG4, 14-amino-3,6,9,12-tetraoxatetradecan-1-oic acid) and DT12 ([(PEG6)Lys^7]DT1; PEG6, 2,5,8,11,14,17-hexaoxa-nonadecan-19-oic acid) from PiChem Forschungs- und Entwicklungs GmbH (Raaba-Grambach, Austria), comprising purity via HPLC analysis and MALDI-TOF data is summarized in Table S1.

Table S1. Analytical data for DT10, DT11 and DT12.[a]

	HPLC				MW [c] calcd	MW found [d], m/z
	t_R (min)		% Purity			
DT10	12.5 [a]	26.6 [a]	>95 [b]	>97 [b]	1291.6	1292.2
DT11	12.7 [a]	27.1 [a]	>95 [b]	>98 [b]	1526.9	1526.8
DT12	9.7 [a]	22.0 [a]	>95 [b]	>97 [b]	1425.8	1425.0

[a] A Nucleosil C18 (5 µm, 4 mm × 150 mm) column (MACHEREY-NAGEL GmbH & Co. KG; Dueren, Germany) was eluted at 1 mL/min flow rate with the following gradient: 90%A/10%B to 10%A/90%B in 30 min, UV trace at 215 nm; A: 0.1% TFA, B: 0.1%TFA in MeCN. [b] A Symmetry Shield RP-18 (5 µm, 4.6 mm × 150 mm) cartridge column (Waters, Vienna, Austria) was eluted at a 1 mL/min flow rate with the following linear gradient (system 2): from 100%A/0%B to 60% A/40% B in 40 min; A = 0.01% TFA and B = MeCN – UV trace at 220 nm; [c] average mass; [d] verification on MALDI TOF mass spectrometry.

Radioanalytical data for [^{99m}Tc]Tc-DT10, [^{99m}Tc]Tc-DT11 and [^{99m}Tc]Tc-DT12

Analytical data for the new [^{99m}Tc]Tc-DT1 mimics: [^{99m}Tc]Tc-DT10, [^{99m}Tc]Tc-DT11 and [^{99m}Tc]Tc-DT12 in two HPLC systems is summarized in Table S2.

Table S2. Radioanalytical data for [^{99m}Tc]Tc-DT10, [^{99m}Tc]Tc-DT11 and [^{99m}Tc]Tc-DT12.[a]

	HPLC			
	t_R (min)		% Purity	
[^{99m}Tc]Tc-DT10	13.4 [a]	28.2 [b]	>99 [a]	>99 [b]
[^{99m}Tc]Tc-DT11	13.7 [a]	28.7 [b]	>98 [a]	>99 [b]
[^{99m}Tc]Tc-DT12	11.8 [a]	23.2 [b]	>99 [a]	>98 [b]

A Waters Symmetry Shield RP-18 (5 µm, 4.6 mm × 150 mm) cartridge column (Waters, Vienna, Austria) was eluted at a 1 mL/min flow rate with either [a] system 1: from 100%A/0%B to 40%A/60%B in 30 min; A = 0.01% TFA and B = MeCN – gamma trace; or [b] system 2: from 100% A/0% B to 60% A/40% B in 40 min – gamma trace.
